# The Chinese version of the tendency to stigmatize epidemic diseases scale: a translation and validation study

**DOI:** 10.3389/fpsyt.2024.1415404

**Published:** 2024-09-03

**Authors:** Xin Wang, Yuecong Wang, Yuanhui Ge, Yuxiu Liu, Riyu Niu, Zhengxiang Guo, Dongfang Ge

**Affiliations:** ^1^ Department of Nursing, Huaian Hospital of Huaian City, Huaian, China; ^2^ Department of Nursing, Huzhou University, Huzhou, China; ^3^ Department of Nursing, Jinzhou Medical University, Jinzhou, China; ^4^ Department of Infectious Diseases, Huaian Hospital of Huaian City, Huaian, China

**Keywords:** infectious disease, stigma, translation, intercultural adaptation, psychometric assessment

## Abstract

**Objective:**

To translate the Tendency to Stigmatize Epidemics Diseases Scale (TSEDS) into Chinese and to evaluate its psychometric properties.

**Methods:**

Translation and cross-cultural adaptation using the Brislin translation model, and pre-testing to form a Chinese version of TSEDS. A total of 434 adults participated in the study and the TSEDS were measured using the critical ratio method, Pearson correlation analysis, retest reliability, content validity, structural validity, and concurrent validity.

**Results:**

The Chinese version of the TSEDS scale contains 27 items in 5 dimensions, including structural stigma, perceived stigma, organizational stigma, internalized stigma, and social stigma. The average content validity index of the scale was 0.975. The goodness of fit index (χ2/df= 1.981, RMSEA = 0.067, CFI= 0.930, IFI = 0.931, TLI = 0.922) indicated a good model fit. The Cronbach’s alpha coefficient was 0.962 and the dimensionality ranged from 0.882 to 0.928. The retest reliability was 0.912.

**Conclusion:**

The Chinese version of TSEDS has good reliability and validity, which can be used to assess the epidemiological stigma tendency of Chinese adults.

## 1 Introduction

Infectious diseases are diseases that arise from infection of the human body by pathogenic microorganisms such as viruses, bacteria, fungi, and parasites such as protozoa and worms, which are contagious and can cause epidemics under certain conditions (1). They are the most serious category of diseases that endanger human health and lives (2). It is estimated that approximately 60 million people die globally each year, with at least 25% of these deaths due to infectious diseases (3, 4). Infectious diseases are characterized by complex routes of transmission, high infectiousness, widespread prevalence, and high morbidity (5). An infectious disease is considered an epidemic when it spreads quickly and impacts a large population; prevention and control of this disease is one of the most crucial public health concerns in the world. In 1980, the World Health Organization (WTO) declared the eradication of smallpox, but new threats soon emerged. The AIDS epidemic began in 1981, and the severity of tuberculosis increased with it (6). In 2002, an outbreak of contagious atypical pneumonia (SARS) occurred in Guangdong, China, and quickly spread around the world, with a fatality rate of up to 10% (7). Middle East Respiratory Syndrome (MERS), which broke out in Saudi Arabia in 2012 and has since spread to more than 20 countries worldwide, has a mortality rate of up to 34% (8). To this day, COVID-19 continues to spread, to the detriment of human life, property, health, and safety, and to new challenges to global economic development (9). The WTO states that infectious diseases need to be closely monitored to ensure prevention, promote early detection, and reduce further transmission. The public is prone to prejudice against people with infectious diseases, and patients themselves can have negative psycho-emotional reactions. It has been found that when epidemics associated with infectious diseases such as COVID-19 (10), syphilis (11), AIDS (12), and tuberculosis (13)occur, patients develop different characteristics of disease stigma.

Stigma, also known as “stigma feelings”, refers to the negative emotional reactions that the experiences patients have due to their illness (14). It was first introduced into the field of psychology by American sociologist Goffman in 1963 (15). Stigma is a social phenomenon that occurs when individuals, usually those in groups with low self-esteem, are discriminated against because of their medical condition, leading to stereotyping, labeling, isolation, and lowered status (16). Chinese research population on disease stigma has focused on breast cancer (17), epilepsy (18), and psychiatric disorders (19). Some scholars have also explored the stigma associated with some chronic infectious diseases, such as the fact that stigma can lead to a decrease in self-esteem and self-confidence among AIDS patients, which seriously affects their quality of life (20). The more TB-related stigma, the higher the chance of patients having depressive symptoms (21). Hepatitis-B infected people have different degrees of stigma, which seriously affects their interpersonal relationships and job search (22). Stigma can lead to deterioration of physical and mental health, such as anxiety, depression, mental and emotional distress, and reduced quality of life. High levels of stigma have a significant impact on health, with patients avoiding healthcare services, thereby increasing the transmission of infectious diseases (23).

At present, China mostly uses scales for specific infectious diseases, such as the Chinese version of the AIDS Perceived Discrimination Scale (24), the Tuberculosis Stigma Perception Scale (25), and the Discrimination Measurement Scale for People with Chronic Hepatitis B Virus (26). However, these scales are all developed for specific infectious diseases and are difficult to fully reflect the stigmatization experienced by adults in different epidemic contexts. In view of the frequent occurrence of global epidemics and their widespread psychosocial impact, there is an urgent need for a non-disease-specific scale that can be used across cultural and epidemic contexts to accurately assess the extent of epidemic stigmatization. Sevim et al. developed the Tendency to Stigmatize Epidemic Diseases Scale(TSEDS) in 2014, the accuracy and reliability of these findings have been validated in a Turkish adult population, which is essential for controlling epidemics and protecting public health and safety (27). The TSEDS scale is a new, non-disease-specific measurement tool that can be used in different epidemics to help healthcare professionals understand epidemic-related social emotions and behaviors and to develop policies to reduce stigma during epidemics to provide rapid and timely interventions. Therefore, translating the TSEDS into Chinese and conducting cultural adaptation verification not only fills the gap in this field in China, but also provides strong support for the formulation of public health policies and the implementation of rapid intervention measures. As suggested by the original authors, TSEDS can be validated for applicability by conducting reliability and validity studies in different countries and cultural contexts.

Therefore, the aim of this study was to translate the English version of the TSEDS into Chinese with cross-cultural adaptation, to assess the psychometric properties of the TSEDS Chinese version in a Chinese community-based adult population. This has not only enriched the theoretical framework for the study of epidemic stigma in China, but also provided a scientifically valid measurement tool for subsequent studies. To more systematically assess the status of epidemiological stigma in China and to provide a rational basis for the development of effective interventions. By applying the TSEDS in different epidemic contexts, we can better understand the psychosocial mechanisms of epidemic stigma and provide empirical evidence for formulating more accurate and effective public health intervention strategies. In addition, this study will promote international exchanges and cooperation in the field of epidemiological stigma research to jointly address global public health challenges.

## 2 Methods

### 2.1 Study design and participants

This study was a cross-sectional study. A questionnaire survey was conducted from February to March 2024 using a convenience sampling method among adults in five communities in Huaian City, Jiangsu Province, China. There are 115 communities in the city of Huaian, in order to increase the representativeness of the sample and the accuracy of the research, we selected five communities with relatively active community activities and a relatively complete organizational structure for sampling. These communities are not only evenly distributed geographically, covering different areas of Huai’an City, but also exhibit a certain diversity in socio-economic status, population density, and residents’ health status. The selection of these communities helps us to better understand the medical behavior and psychological status of adults in Huai’an City during the COVID-19 outbreak. As for the respondents, we selected the closest and most accessible eligible adults to ensure that we collected as much valid data as possible in the limited time available. Inclusion criteria: (1) aged 18-59 years; (2) receiving inpatient or outpatient treatment during the COVID-19 epidemic (3) mentally alert and able to understand the questionnaire; (4) informed consent and voluntary participation in this study. Exclusion criteria: people with severe mental illness.

### 2.2 Instruments

#### 2.2.1 Questionnaire on general demographic characteristics

The study was designed according to the content and purpose of the study, including age, gender, education level, average monthly income, marital status, and treatment methods during the COVID-19 epidemic.

#### 2.2.2 Tendency to stigmatize epidemic diseases scale

The scale was developed by Sevim (27) to assess the level of stigma among adults during the epidemic. The scale consists of 27 items and 5 dimensions: structural stigma (7 items), perceived stigma (6 items), organizational stigma (3 items), internalized stigma (6 items), and social stigma (3 items). A Likert scale with 5 points ranging from 1 (strongly disagree) to 5 (strongly agree) was used. Total scores ranged from 27 to 135, with higher scores indicating a greater tendency to epidemiological stigma. The original scale had a Cronbach’s α coefficient of 0.88.

#### 2.2.3 Self-esteem scale

The scale was developed by Rosenberg (28) and the Chinese version by Ji et al. in 1993 (29). It is one of the more influential instruments for measuring an individual’s level of self-esteem. Contains 10 entries on a single dimension using a 4-point Likert scale from 1 (completely disagree) to 4 (completely agree). Total scores range from 10-40, where higher scores represent greater levels of individual self-esteem. The Cronbach’s α coefficient for SES was 0.900, and for the scale in this research, it was 0.873. SES was used to measure concurrent validity.

### 2.3 Procedures

#### 2.3.1 Translation and cultural adaptation

After getting approval from the original author, Professor Sevim, via email, the original scale was translated as well as cross-culturally adapted to creat the Chinese version of the TSEDS, strictly following the Brislin Translation Model (30).

Positive translation of the questionnaire: the English translation of TSEDS was done independently by two nursing graduate students who had passed CET-6 and whose mother tongue was Chinese, and the results of the two translations were synthesized by a nursing psychology faculty member, who formed the Chinese version of TSEDS-1 with the unified opinion of the group.

Back-translation of the questionnaire: Two professors from the English Department with no medical background and no previous exposure to the scale were invited to back-translate the TSEDS-1 into Chinese, and members of the group summarized the two back-translated versions of the scale, which were discussed to form the Chinese version of the TSEDS-2.

The questionnaire was adapted for cross-cultural use by consulting eight experts: three specializing in epidemiology, three in nursing, and two in psychology. Four of them had PhDs and four had master’s degrees. A Likert 4-point scale was used to score the clarity of semantic expression, the relevance of the content of the entries, and the degree of compliance with the cultural context of each entry of the scale and to propose relevant modifications, resulting in the Chinese version of the TSEDS-3.

Pre-survey: 30 adults were invited to participate in a survey to validate the clarity and comprehension of the translated scale items. The results indicated that the scale was easy to understand and easy to fill in (filling in time was about 5 min), thus resulting in the final Chinese version of the TSEDS.

#### 2.3.2 Data collection

Before data collection, all researchers involved were uniformly trained to ensure that everyone was familiar with the questionnaire questions and their meanings, as well as the steps and precautions for unified data collection. Questionnaire collection was conducted in five communities to clarify the inclusion criteria of participants and to minimize potential bias by ensuring that participants were evenly distributed by age and gender. The researcher explained the content, purpose, and significance of the questionnaire to the participants before handing it out, and distributed the paper questionnaire on the spot. The average time to fill out the questionnaire was determined by pre-testing to be about 10 minutes, and on-site supervision was conducted to ensure that each participant had enough time to carefully fill it out. The survey was conducted anonymously to protect participants’ privacy and encourage them to provide truthful and objective responses. Only the personal contact information of 30 adults was retained so that a second survey could be conducted two weeks later to measure the retest reliability of the scale. The questionnaire was collected on site immediately after completion to ensure data integrity and timeliness.

According to the criteria proposed by Kendall (31), a sample size of at least 5-10 times the scale items and a minimum of 200 cases is required for validated factor analyses (CFA) (32). A total of 27 items in the Chinese version of TSEDS were used in this research. Considering a 10% invalid sample size, 450 adults were enlisted for study participation, resulting in the recovery of 434 questionnaires after eliminating invalid ones, yielding a valid recovery rate of 96.44%.

## 3 Data analysis

### 3.1 Statistical analysis

The data of this study was entered by two persons using Excel software and statistically analyzed using SPSS 27.0 and AMOS 24.0. Demographic characteristics were analyzed using descriptive statistics (mean ± standard deviation of continuous data, frequencies, and percentages of demographic characteristics). When the skewness and kurtosis values of the items were within ±2 (33), the data were considered normally distributed. This study used critical ratio method and correlation coefficient method to measure item analysis, internal consistency reliability and rest-retest reliability to measure reliability analysis, and correlation validity, construct validity, and concurrent validity to measure validity analysis.

### 3.2 Item analysis

#### 3.2.1 Critical ratio method

An independent samples t-test was employed to separate the top 27% (high group) and the bottom 27% (low group) of the Chinese TSEDS total scores, which were arranged from low to high. The item was kept when the CR value was >3.0 and *p* < 0.05 (34).

#### 3.2.2 Correlation coefficient method

The correlation coefficients of the items with the scale’s overall score were analyzed, and the items with correlation coefficients <0.4 or no significant difference were excluded. The correlation coefficients between the items and the total score were computed, and the Cronbach’s α values follow deleting the items. If the Cronbach’s α value increased after the deletion of the item, the item could be eligible for deletion (35).

### 3.3 Reliability analysis

#### 3.3.1 Internal consistency reliability

Corrected item-total correlation and Cronbach’s α coefficient were used. Acceptable Cronbach’s α coefficients were defined as ≥0.70 and a standardized adjusted item-total correlation value of >0.3 (35).

#### 3.3.2 Test-retest reliability

Thirty participants who completed the first test were randomly selected for a retesting two weeks after the initial questionnaire. Correlation analyses of the results of the two surveys were conducted using Pearson correlation to test the retest reliability of the scale. The scale reliability was considered to be good, when the retest reliability was >0.75 (35).

### 3.4 Validity analysis

#### 3.4.1 Content validity

A 4-point Likert scale was used to assess the application, relevance, and completeness of the concepts, standards, and semantics of the Chinese version of TSEDS by eight experts with relevant experience: not relevant (one point), low relevance (two points), medium relevance (three points), and strong relevance (four points). According to the Delphi method, when the Scale-Content Validity Index (S-CVI) at the scale level is >0.900 and the Item-Content Validity Index (I-CVI) at the item level is >0.780, the scale has strong content validity (36).

#### 3.4.2 Construct validity

To explore the underlying factor structure of the translated questionnaire, the structural validity of the TSEDS was examined using validation factor analysis (CFA) and exploratory factor analysis (EFA). 434 adults in total, with 217 participants in each group, were randomly assigned to the EFA and CFA groups.

In sample 1 (n=217), exploratory factor analysis (EFA) was carried out. When the Bartlett sphericity test was statistically significant (*p* < 0.05) and Kaiser-Meyer-Olkin (KMO) was >0.60, factor analysis was suitable (37). The orthogonal rotation approach and principal component analysis were utilized to extract common elements with eigenvalues greater than 1.

In Sample 2 (n=217), a validation factor analysis (CFA) was performed using AMOS to confirm that the model structure and the factor structure under investigation were consistent. The maximum likelihood approach was used for estimation, and the following metrics were used to calculate the model fit indices: chi-square degrees of freedom (χ2/df) < 3.0, comparative fit index (CFI) > 0.9, index of value-added fit (IFI) > 0.9, Tucker-Lewis index (TLI) > 0.9, and root mean square of the error of approximation (RMSEA) < 0.08 indicating that the model fit was within an acceptable range (38).

#### 3.4.3 Concurrent validity

SES was used as a calibration tool in this study to evaluate the correlation coefficients between the scale scores and other variables since it is commonly used and has strong reliability and validity. The degree of correlation between variables can be assessed based on the correlation coefficient’s magnitude and absolute value. It is best when the correlation coefficient’s absolute value falls between 0.4 and 0.7 (39).

### 3.5 Ethical approval

The research was granted approval by Jinzhou Medical University’s Ethics Committee (consent number: JZMULL20240708), and and every subject filled out an informed consent form.

## 4 Results

### 4.1 Demographics and sample characteristics

A total of 434 adults were enrolled, of whom 225 (51.8%) were female; 130 (30.0%) were aged 18-29 years; 293 (67.5%) were married; 293 (67.5%) had a university degree or higher; 184 (42.4%) had an average monthly income of $3,000-$5,000; and 371 people (85.5%) chose outpatient treatment during the COVID-19 pandemic. See **Table 1** for details.

**Table 1:** Demographic characteristics of participants (N = 434).

	Variable	N	%
Gender	Male	209	48.2
	Female	225	51.8
Age group (years)	18-29	130	30.0
	30-39	112	25.8
	40-49	106	24.4
	50-59	86	19.8
Marital status	Single	125	28.8
	Married	293	67.5
	Divorced or widowed	16	3.7
Educational level	Primary school and below	52	12.0
	Junior and senior high schools	89	20.5
	College degree or above	293	67.5
Monthly income (yuan)	<3000	153	35.3
	3000-5000	184	42.4
	>5000	97	22.4
Treatment during the COVID-19 pandemic	Outpatient treatment	371	85.5
	Receive hospital treatment	63	14.5

### 4.2 Item analysis

Each item’s CR value ranged from 11.779 to 26.805 (>3.0) according to the critical ratio method, all of which were statistically significant (*p* < 0.001). All correlation coefficients were greater than 0.4, and the range of correlations between the items and the scale’s overall score was 0.588 to 0.773 (*p* < 0.01). Furthermore, the total Cronbach’s α coefficient for the scale decreased upon the removal of any item. These findings show that all 27 elements were kept from the Chinese version of the TSEDS entries, which demonstrated good discrimination. See **Table 2** for details.

**Table 2:** Score comparison between high-score and low-score groups, item analysis (N=434).

Item	Low-score group (n=117),mean (SD)	High-score group(n=129),mean (SD)	Critical ratio	Item-total correlation	Cronbach’s alpha if item delete
1	2.60(0.492)	3.83(0.663)	16.400^**^	0.624^**^	0.961
2	1.97(0.804)	3.86(0.778)	18.693^**^	0.687^**^	0.961
3	2.23(0.792)	3.91(0.718)	17.403^**^	0.620^**^	0.961
4	2.52(0.794)	3.98(0.696)	15.299^**^	0.650^**^	0.961
5	2.58(0.757)	4.08(0.714)	15.953^**^	0.665^**^	0.961
6	1.93(0.751)	3.83(0.782)	19.371^**^	0.723^**^	0.960
7	2.26(0.700)	4.05(0.653)	20.672^**^	0.724^**^	0.960
8	1.88(0.790)	4.09(0.643)	23.955^**^	0.769^**^	0.960
9	1.91(0.841)	4.06(0.693)	22.024^**^	0.747^**^	0.960
10	2.09(0.847)	4.03(0.695)	19.580^**^	0.699^**^	0.961
11	1.73(0.567)	3.92(0.703)	26.805^**^	0.767^**^	0.960
12	2.19(0.642)	4.16(0.618)	24.466^**^	0.732^**^	0.960
13	2.14(0.681)	4.08(0.853)	19.797^**^	0.735^**^	0.960
14	1.77(0.747)	3.75(0.848)	19.371^**^	0.745^**^	0.960
15	1.60(0.631)	3.17(0.945)	15.479^**^	0.721^**^	0.960
16	1.37(0.581)	3.34(0.940)	20.006^**^	0.744^**^	0.960
17	1.54(0.550)	3.49(0.876)	21.108^**^	0.773^**^	0.960
18	1.55(0.549)	3.53(0.820)	22.426^**^	0.759^**^	0.960
19	1.72(0.668)	3.84(0.908)	20.981^**^	0.759^**^	0.960
20	1.69(0.533)	3.63(1.008)	19.065^**^	0.766^**^	0.960
21	2.44(1.086)	3.81(0.650)	11.779^**^	0.588^**^	0.961
22	1.62(0.628)	3.26(1.099)	14.541^**^	0.736^**^	0.960
23	1.59(0.645)	3.36(0.865)	18.337^**^	0.708^**^	0.960
24	1.95(0.797)	3.83(0.802)	18.427^**^	0.705^**^	0.960
25	2.26(0.767)	3.64(0.918)	12.825^**^	0.658^**^	0.961
26	1.83(0.673)	3.26(0.886)	14.293^**^	0.674^**^	0.961
27	1.92(0.672)	3.33(0.953)	13.434^**^	0.645^**^	0.961

^**^ *p*<0.01.

### 4.3 Reliability analysis

The Cronbach’s α coefficients for the dimensions varied from 0.882 to 0.928, while the total Cronbach’s α coefficient was 0.962. All of the items had corrected item-total correlations that were greater than 0.3, ranging from 0.553 to 0.750. A follow-up test was conducted on thirty persons after two weeks to assess the scale’s reliability, the retest reliability was 0.912. The participants’ mean (SD) scores for each item in the TSEDS’s Chinese version are displayed in **Table 3**. These had a normal distribution based on skewness and kurtosis.

**Table 3:** Mean (SD) scores with skewness and kurtosis, reliability analysis (N=434).

Factor	Item	Mean(SD)	Skewness	Kurtosis	Corrected item-total correlation	Cronbach's α coefficient
Factor 1	1	3.29(0.784)	-0.045	-0.598	0.599	0.888
	2	2.94(1.045)	0.013	-0.736	0.658	
	3	3.08(0.997)	-0.162	-0.728	0.587	
	4	3.35(0.911)	-0.257	-0.226	0.622	
	5	3.34(0.918)	-0.212	-0.278	0.638	
	6	2.99(1.028)	-0.148	-0.536	0.697	
	7	3.25(0.970)	-0.262	-0.393	0.699	
Factor 2	8	3.01(1.183)	-0.026	-0.990	0.742	0.928
	9	2.97(1.160)	0.032	-0.979	0.720	
	10	3.16(1.100)	-0.151	-0.824	0.669	
	11	2.85(1.162)	0.175	-0.864	0.741	
	12	3.27(1.051)	-0.249	-0.624	0.706	
	13	3.15(1.101)	0.112	-0.980	0.708	
Factor 3	14	2.80(1.083)	0.109	-0.925	0.719	0.916
	15	2.41(0.953)	0.406	-0.394	0.697	
	16	2.39(1.087)	0.421	-0.671	0.718	
	17	2.49(1.033)	0.435	-0.534	0.750	
	18	2.44(1.048)	0.474	-0.675	0.736	
Factor 4	19	2.67(1.171)	0.409	-0.847	0.732	0.905
	20	2.61(1.048)	0.528	-0.370	0.743	
	21	3.23(1.006)	-0.697	-0.408	0.553	
	22	2.33(1.029)	0.861	0.086	0.710	
	23	2.42(1.033)	0.407	-0.744	0.681	
	24	2.96(1.112)	-0.080	-0.970	0.675	
Factor 5	25	3.02(0.957)	0.054	-0.583	0.630	0.882
	26	2.47(0.932)	0.370	-0.365	0.647	
	27	2.59(0.968)	0.434	-0.376	0.615	

### 4.4 Validity analysis

#### 4.4.1 Content validity

The findings demonstrated that the S-CVI was 0.95 and the I-CVI ranged from 0.84 to 1.00, indicating that the questionnaire’s content validity was good.

#### 4.4.2 Construct validity

**Figure 1:**
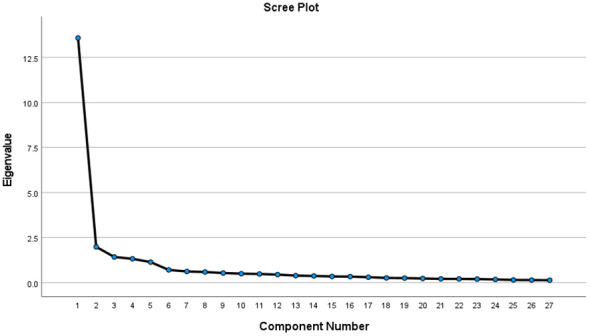
Screen plot of exploratory factor analysis for the Chinese version of the TSEDS (n = 217).

Exploratory factor analysis (EFA): The Bartlett sphericity test approximate chi-square for this study was 4555.604 (*p* < 0.001) and the KMO value was 0.947 (> 0.6), making it suitable for factor analysis. After applying the data to PCA with maximum variance orthogonal rotation, five factors with eigenroots > 1 were extracted, with the same number of factors as in the original scale. The cumulative variance contribution was 72.045%, with each item loading value > 0.4. The loading matrices of the factors are shown in **Table 4**. The gravel plot, which shows a weaker decreasing trend after point 5, helps to further illustrate the structure of the five variables. In **Figure 1**, the gravel plot is displayed.

**Table 4:** Factor loadings of the TSEDS (N = 217).

Item	Factor 1	Factor 2	Factor 3	Factor 4	Factor 5
10	0.774				
9	0.756				
12	0.753				
8	0.747				
11	0.720				
13	0.671				
2		0.787			
7		0.723			
4		0.693			
5		0.677			
3		0.590			
1		0.587			
6		0.546			
16			0.774		
15			0.767		
18			0.763		
17			0.700		
14			0.562		
21				0.773	
23				0.728	
19				0.647	
24				0.643	
22				0.626	
20				0.588	
27					0.841
26					0.788
25					0.757

**Figure 2:**
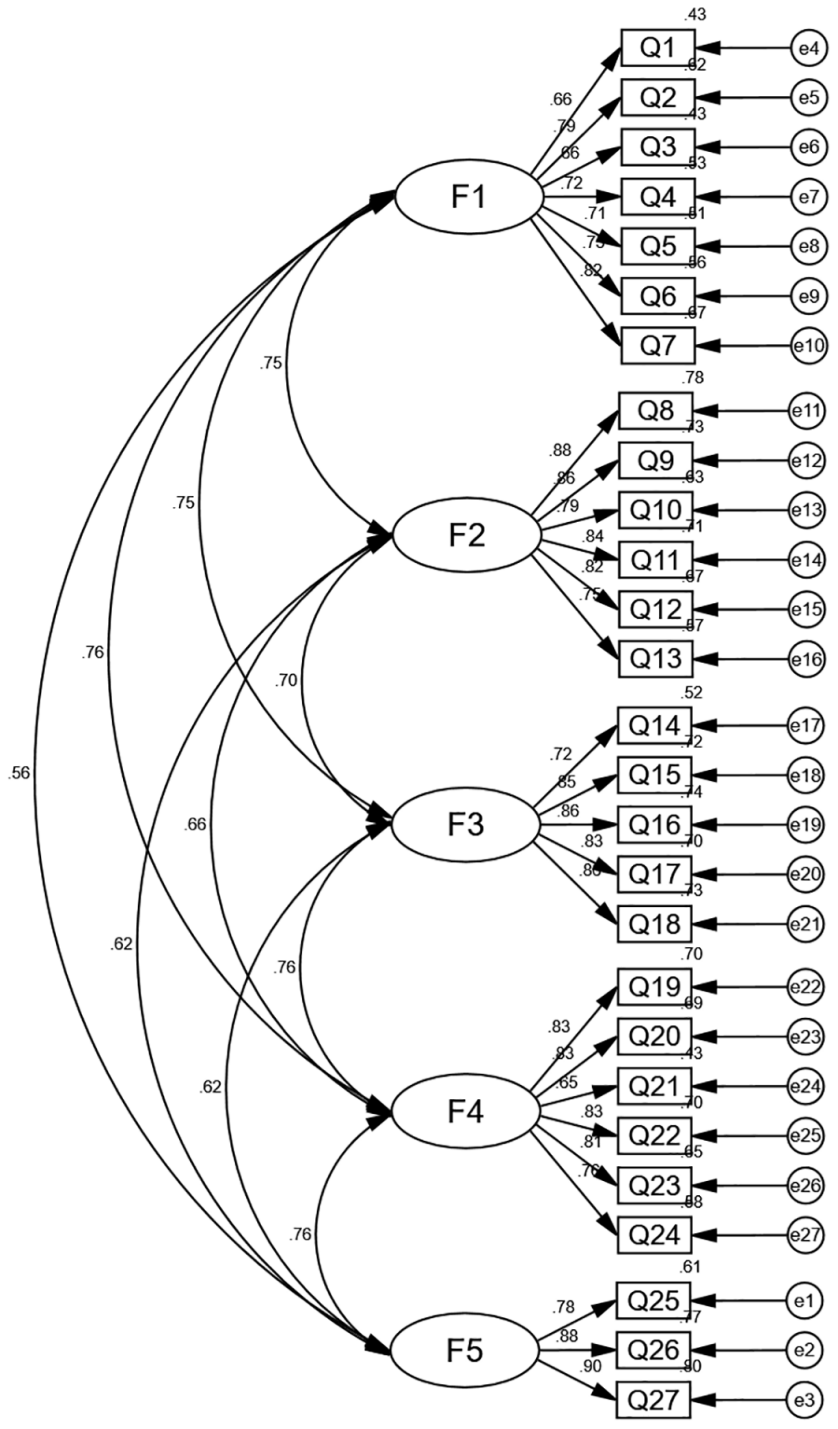
Standardized five-factor structural model of the Chinese version of the TSEDS (n = 217). F1: Structural stigma; F2: Perceived stigma; F3: Organizational stigma; F4: Internalized stigma; F5: Social stigma.

Confirmatory factor analysis (CFA): the validation results showed good results in this study. The indicators’ values are as follows: χ2/df = 1.981, CFI = 0.930, TLI = 0.922, IFI = 0.931, and RMSEA = 0.067. The CFA results are shown in **Figure 2**.

#### 4.4.3 Concurrent validity

Between the SES total score and the Chinese version of the TSEDS total score, the absolute value of the Pearson correlation coefficient was 0.579 (*p* < 0.01), a statistically significant difference indicating a high degree of correlation.

## 5 Discussion

One of the reasons why fewer studies on epidemic disease stigma have been reported in China is the dearth of measurement instruments for epidemic disease stigma tendencies. To scientifically and validly assess adults’ propensity to feel stigma during the epidemic, we translated the TSEDS from English into Chinese and conducted comprehensive psychometric analyses, including item analyses, reliability analyses, and validity analyses, on 434 adults. The scale applied for the first time to the Chinese population, has good reliability and validity, helping to identify the tendency of adults to feel stigma during the epidemic and prompting healthcare professionals to give timely and targeted interventions to reduce patients’ negative emotions and improve their mental and physical health.

One of the most important phases in the process of revising the scale is item analysis, as it contributes to the quality of the test items (34). The CR values of the 27 items in this study were all >3.0 (*p* < 0.001), demonstrating a significant level of scale discrimination. Every item’s correlation coefficient and the scale’s overall score were all more than 0.588, showing a medium to high correlation. In addition, after removing the items, the translated scale’s Cronbach’s α coefficient dropped, demonstrating a high degree of internal consistency and a strong connection between the components. It suggests that all 27 items in the TSEDS’s Chinese translation have good discriminability and can better reflect the tendency of disease stigma during the adult epidemic, and all of them can be retained.

Reliability refers to the stability and consistency of the results measured by a scale, and the greater the reliability of a scale, the smaller its standard error of measurement. The Cronbach’s α coefficients of the Chinese version of the TSEDS and the dimensions in this study meet the reference standard, indicating that the scale has high internal consistency reliability (35). Compared with the original scale, the Cronbach’s α coefficients of the Chinese version of the TSEDS are higher than those of the original scale (27). This may be related to the fact that China has a large population base and the the rapid spread of the epidemic is more likely to cause stigmatization. Every item’s correlation coefficient with the overall score was greater than 0.4, indicating good internal consistency of the scale (35). In the meantime, the retest reliability was strong, demonstrating the TSEDS’s longitudinal stability in Chinese. Therefore, the TSEDS in Chinese has good reliability.

Validity is the validity or accuracy of a scale’s findings (36). The content validity, structural validity, and concurrent validity of the Chinese version of the TSEDS were assessed in this study. Eight experts from the fields of epidemiology, nursing, and psychology were invited to evaluate the content’s validity. The values of the I-CVI and the S-CVI were within a reasonable range (36). This suggests that the items of the Chinese version of the TSEDS can effectively respond to the tendency to feel stigma during the adult epidemic. The degree to which a scale is integrated with the theoretical or conceptual framework that forms its basis is reflected in its structural validity (37). Five metric factors were recovered from the EFA without removing any entries, and the entries for every dimension were consistent with the original scale, with a cumulative variance contribution of 72.045%. Five factors were shown in the Chinese version of the TSEDS: structural stigma, perceived stigma, organizational stigma, internalized stigma, and social stigma. The translation scale’s structure was further verified in this study using CFA, and the fitting index met the ideal criteria, demonstrating the scale’s strong structural validity (38). In addition, concurrent validity analyses indicated that there was a correlation between the Chinese version of the TSEDS and the SES scores (*p* < 0.05), suggesting that the scale has good concurrent validity.

The first factor is structural stigma, which emphasizes inequity in health services. Every member of society, regardless of his or her occupation, income, etc., should have equal access to the health services that he or she needs, such as prevention, medical treatment, and health care. However, the problem of inequity in public health services in the field of infectious diseases is still relatively serious at present, and the general public is reluctant to share public transport with patients with infectious diseases and would like to keep a distance from them, indicating that the public health service system needs to be strengthened to ensure that all members of society have equal access to the preventive, medical, and health services they need, and to promote public education in order to increase social understanding and acceptance of those affected by the epidemic, and reduce unnecessary discrimination and exclusion. The second factor is perceived stigma, which is the individual’s feelings of shame, including negative emotions such as embarrassment, guilt, loneliness, resentment, and fear. These negative emotional impacts may have important implications for the effectiveness of diagnosis and treatment of diseases in people with infectious diseases and may lead to delays in healthcare seeking, resulting in delays in diagnosis and initiation of treatment, increasing the contagiousness of the disease, and affecting the health of the individual as well as that of others (40). The resultant perceived stigma may also lead to the avoidance of social activities, and ultimately to social isolation. Medical institutions need to carry out mental health education activities to help infectious disease patients and their families correctly understand and cope with the negative emotions brought by the disease, as well as enhance their psychological resilience. They should establish a professional psychological counseling and support system to provide timely psychological assistance and counseling for patients with infectious diseases. Medical staff are encouraged to pay attention to the psychological needs of patients in the treatment process and provide comprehensive medical and psychological support.

The third factor is organizational stigma, which refers to the issues that may be experienced in places of public institutions such as hospitals, apartment buildings, and lifts. Some members of the public do not want to share the public sphere with people with infectious diseases, and public rejection and discrimination may increase the negative experiences and experiences of people with infectious diseases, resulting in avoidance of psychology and behaviors that cause a range of mental health problems. Health management and publicity in public places should be strengthened to raise public awareness of epidemic prevention and control. A non-discrimination policy should be developed and implemented in public places to ensure equal access to public facilities and services for persons affected by or living with epidemics. Public institutions should be encouraged and supported to carry out care activities for these individuals, in order to reduce their feelings of isolation and exclusion. The fourth factor is internalized stigma, whereby the stigmatized individual accepts and rationalizes the negative perceptions that others have of the disease. Individuals with an infectious disease close themselves off psychologically and operationally, fail to accept themselves well evaluate themselves positively, and gradually alienate themselves from their loved ones and friends. Communities and medical institutions can provide mental health education and counseling services to help individuals affected by or living with epidemics develop a positive sense of self-identity and self-worth. Encourage patients to participate in social activities, keep in touch with friends and family, and thereby reduce psychological estrangement and loneliness. Develop patient support groups or online communities where patients can share experiences, encouragement, and support with each other. The fifth factor is social stigma, which manifests the intolerant behaviors and attitudes of society experienced by individuals with infectious diseases. Increase public understanding and empathy for those affected by the epidemic, and reduce discrimination and prejudice through media campaigns and educational activities. Promote social inclusion and acceptance, and encourage all sectors of society to provide support and help for epidemic patients. Develop and implement anti-discrimination laws and regulations to protect the legitimate rights and interests of epidemic patients from infringement.

The Chinese version of the TSEDS has been analyzed for item analysis, validity, and reliability. It can be used to measure the propensity to feel stigmatized during epidemics among Chinese adults. This scale is good for encouraging the development of policies to reduce stigma during epidemics, as well as providing healthcare professionals with an understanding of epidemic-related social emotions and behaviors, and timely interventions to reduce the problem of stigma.

This study does, however, have certain limitations. Firstly, the study’s participants were from five communities in Huaian, and therefore not fully representative of the diversity of Chinese adults. Further expansion of the sample size is needed to improve the applicability of the scale in the future. Furthermore, as the TSEDS is a self-report measure, bias in the results is inevitable.

## 6 Conclusion

The Chinese version of the TSEDS has clear entries and consists of five factors: structural stigma, perceived stigma, organizational stigma, internalized stigma, and social stigma. It has good validity, reliability, and psychometric characteristics. It can be used as an effective tool for the epidemiological stigma tendency of Chinese adults.

## Data Availability

The original contributions presented in the study are included in the article/Supplementary Material. Further inquiries can be directed to the corresponding authors.
