# Supplementary material for: The Chinese version of the tendency to stigmatize epidemic diseases scale: a translation and validation study
Source: Front Psychiatry. 2024 Sep 3;15:1415404. doi: 10.3389/fpsyt.2024.1415404 (PMC11406073; doi:10.3389/fpsyt.2024.1415404)
Supplement: Supplementary file 1 [file Table1.docx]

**Epidemiological Stigma Surveys**

Hello! We conducted this survey to find out your views on epidemics such as COVID-19, influenza, chickenpox, tuberculosis, etc. Please tick "√" in the corresponding box according to the actual situation. All data is for research only. Please answer according to the actual situation. Thanks again for your cooperation!

**Demographic characteristics of participants**

1. Gender：①Male ②Female
2. Age group (years)：①18-29 ②30-39 ③40-49 ④50-59
3. Marital status：①Single ②Married ③Divorced or widowed
4. Educational level：①Primary school and below ②Junior and senior high schools ③College degree or above
5. Monthly income (yuan)：①＜3000 ②3000-5000 ③＞5000

Treatment during the COVID-19 pandemic**：**①Outpatient treatment ②Receive hospital treatment

**Tendency to Stigmatize Epidemic Diseases Scale（TSEDS）**

| Item | Strongly Disagree | Disagree | Fairly | Agree | Strongly Agree |
| --- | --- | --- | --- | --- | --- |
| 1. People who catch epidemic diseases should have exclusive public transportation | 1 | 2 | 3 | 4 | 5 |
| 1. People who catch epidemic diseases should not have treatments at hospital with other people | 1 | 2 | 3 | 4 | 5 |
| 1. People who catches epidemic diseases should not be using the common areas with their neighbors | 1 | 2 | 3 | 4 | 5 |
| 1. People should be distant from the people who catch epidemic diseases | 1 | 2 | 3 | 4 | 5 |
| 1. People should be afraid of contacting with each other during pandemics | 1 | 2 | 3 | 4 | 5 |
| 1. People who catch epidemic diseases are prevented to enter public building or social venues | 1 | 2 | 3 | 4 | 5 |
| 1. People who catch epidemic diseases are taken an area away from other patients in the hospital | 1 | 2 | 3 | 4 | 5 |
| 1. I would be embarrassed if I catch an epidemic disease | 1 | 2 | 3 | 4 | 5 |
| 1. I would feel guilty if I catch an epidemic disease | 1 | 2 | 3 | 4 | 5 |
| 1. I would feel alone if I catch an epidemic disease | 1 | 2 | 3 | 4 | 5 |
| 1. I would feel resentful due to the reactions of the other people if I catch an epidemic disease | 1 | 2 | 3 | 4 | 5 |
| 1. I would be afraid that people might judge me if I catch an epidemic disease | 1 | 2 | 3 | 4 | 5 |
| 1. I would be afraid that people might not contact me if I catch an epidemic disease | 1 | 2 | 3 | 4 | 5 |
| 1. People who catch epidemic diseases should be isolated from society | 1 | 2 | 3 | 4 | 5 |
| 1. I would not want to take an elevator with healthcare workers | 1 | 2 | 3 | 4 | 5 |
| 1. I would not want to live in the same apartment building with healthcare workers | 1 | 2 | 3 | 4 | 5 |
| 1. Hospitals that allow patients with epidemic diseases should not be never visited | 1 | 2 | 3 | 4 | 5 |
| 1. Healthcare workers who take care of people with epidemic diseases should not enter public areas | 1 | 2 | 3 | 4 | 5 |
| 1. If someone close to me (friends, family member etc.) catches an epidemic disease, I would be contacting them less | 1 | 2 | 3 | 4 | 5 |
| 1. If I catch an epidemic disease, my friends would be more distant. | 1 | 2 | 3 | 4 | 5 |
| 1. If I catch an epidemic disease, I would stop contacting with the people due to their reactions | 1 | 2 | 3 | 4 | 5 |
| 1. If I catch an epidemic disease, I would not enjoy anything | 1 | 2 | 3 | 4 | 5 |
| 1. If I catch an epidemic disease, relationships with my family would be affected | 1 | 2 | 3 | 4 | 5 |
| 1. If I catch an epidemic disease, relationships with my social | 1 | 2 | 3 | 4 | 5 |
| 1. People who catch epidemic diseases would be reluctant to tell to other people | 1 | 2 | 3 | 4 | 5 |
| 1. People who catch epidemic diseases would be outcast from society | 1 | 2 | 3 | 4 | 5 |
| 1. People who catch epidemic diseases would be discriminated from society | 1 | 2 | 3 | 4 | 5 |

**Self-Esteem Scale (SES)**

| Item | Not at all | No | Conforms | Very Conforms |
| --- | --- | --- | --- | --- |
| 1. I feel like I'm a valuable person, at least on the same level as everyone else | 1 | 2 | 3 | 4 |
| 1. I feel I have many good qualities. | 1 | 2 | 3 | 4 |
| 1. When it comes down to it, I tend to feel like a failure. | 1 | 2 | 3 | 4 |
| 1. I can get things done like most people. | 1 | 2 | 3 | 4 |
| 1. I feel I have little to be proud of. | 1 | 2 | 3 | 4 |
| 1. I'm positive about myself. | 1 | 2 | 3 | 4 |
| 1. Overall, I'm happy with myself. | 1 | 2 | 3 | 4 |
| 1. I wish I could earn more respect for myself. | 1 | 2 | 3 | 4 |
| 1. I do feel useless from time to time. | 1 | 2 | 3 | 4 |
| 1. I often think I'm worthless. | 1 | 2 | 3 | 4 |
